# The crosstalk between MYC and mTORC1 during osteoclastogenesis

**DOI:** 10.3389/fcell.2022.920683

**Published:** 2022-08-19

**Authors:** Seyeon Bae, Brian Oh, Jefferson Tsai, Peter Sang Uk Park, Matthew Blake Greenblatt, Eugenia G. Giannopoulou, Kyung-Hyun Park-Min

**Affiliations:** ^1^ Arthritis and Tissue Degeneration Program, David Z. Rosensweig Genomics Research Center, Hospital for Special Surgery, New York, NY, United States; ^2^ Department of Medicine, Weill Cornell Medical College, New York, NY, United States; ^3^ Department of Pathology, Weill Cornell Medical College, New York, NY, United States; ^4^ Biological Sciences Department, New York City College of Technology, City University of New York, Brooklyn, NY, United States; ^5^ BCMB Allied Program, Weill Cornell Graduate School of Medical Sciences, New York, NY, United States

**Keywords:** MYC (c-myc), GADD34 (PPP1R15A), osteoclast (OC), mTORC1 (mechanistic target of rapamycin complex 1), bone resorption

## Abstract

Osteoclasts are bone-resorbing cells that undergo extensive changes in morphology throughout their differentiation. Altered osteoclast differentiation and activity lead to changes in pathological bone resorption. The mammalian target of rapamycin (mTOR) is a kinase, and aberrant mTOR complex 1 (mTORC1) signaling is associated with altered bone homeostasis. The activation of mTORC1 is biphasically regulated during osteoclastogenesis; however, the mechanism behind mTORC1-mediated regulation of osteoclastogenesis and bone resorption is incompletely understood. Here, we found that MYC coordinates the dynamic regulation of mTORC1 activation during osteoclastogenesis. MYC-deficiency blocked the early activation of mTORC1 and also reversed the decreased activity of mTORC1 at the late stage of osteoclastogenesis. The suppression of mTORC1 activity by rapamycin in mature osteoclasts enhances bone resorption activity despite the indispensable role of high mTORC1 activation in osteoclast formation in both mouse and human cells. Mechanistically, MYC induces Growth arrest and DNA damage-inducible protein (GADD34) expression and suppresses mTORC1 activity at the late phase of osteoclastogenesis. Taken together, our findings identify a MYC-GADD34 axis as an upstream regulator of dynamic mTORC1 activation in osteoclastogenesis and highlight the interplay between MYC and mTORC1 pathways in determining osteoclast activity.

## Introduction

Osteoclasts are derived from myeloid lineage cells and regulate bone homeostasis by degrading bone (Teitelbaum, 2000; Ikeda and Takeshita, 2016). Bone resorption by osteoclasts is tightly regulated to maintain skeletal health, as alterations in the formation or activity of osteoclasts results in skeletal diseases, such as osteoporosis (Bi et al., 2017). Macrophage colony-stimulating factor (M-CSF) and receptor activator of nuclear factor kappa-B ligand (RANKL) are key cytokines contributing to osteoclast formation. M-CSF induces receptor activator of nuclear factor kappa-B (RANK) expression and in response to RANKL, those cells induce molecular and metabolic changes that drive osteoclast differentiation and function (Feng and Teitelbaum, 2013; Park-Min, 2019).

The mammalian target of rapamycin (mTOR) is a Serine/Threonine kinase. mTOR forms two distinct complexes, mTOR complex 1 (mTORC1) and mTORC2, which differentially regulate cellular programs. The mTORC1 pathway mainly regulates cell growth, metabolism, and protein synthesis, while mTORC2 controls cell survival (Saxton and Sabatini, 2017). The heterodimeric tuberous sclerosis complex (TSC) 1/2 complex negatively regulates mTORC1 activity by targeting its upstream Rheb GTPase, and the deletion of TSC1 or TSC2 leads to hyperactivation of mTORC1 (Zhang et al., 2003; Huang and Manning, 2008). Notably, the mTOR pathway play important roles in regulating skeletal development and homeostasis by affecting osteoblasts, osteoclasts, and chodrocytes (Chen and Long, 2018). The importance of mTORC1 signaling in osteoclast differentiation has been implicated by pharmacological and genetic modulation (Dai et al., 2017a; Zhang et al., 2017; Hiraiwa et al., 2019). Although mTORC1 activity is dynamically regulated during osteoclast differentiation, its role in osteoclasts remains controversial. Moreover, the mechanisms by which the mTOR pathway is regulated in osteoclasts remain unclear.

MYC is a transcription factor regulating cell proliferation, metabolism, and differentiation (Dang, 1999). The contribution of MYC to physiological and pathological bone loss has been previously highlighted (Bae et al., 2017). RANKL-inducible MYC drives osteoclast differentiation by reprogramming osteoclastogenic pathways and oxidative metabolism *via* direct regulation of nuclear factor of activated T cells, c1 (NFATc1) and estrogen receptor–related receptor α (ERRα), respectively. In addition to NFATc1 and ERR⍺-dependent pathways, multifaced roles of MYC can be supported by as-yet undefined mechanisms (Lorenzo, 2017). Given the importance of MYC in osteoclast biology, the precise mechanisms of MYC’s action in osteoclasts need to be further clarified.

In this study, we identify MYC as an upstream regulator of mTORC1 signaling in osteoclastogenesis. mTORC1 activity is dynamically regulated during human and mouse osteoclast differentiation. Inactivation of mTORC1 in osteoclasts augments their bone-resorbing function, while prolonged activation of mTORC1 limits the bone-resorbing function of osteoclasts. MYC coordinates the dynamic regulation of mTORC1 by regulating GADD34. GADD34 is induced in a MYC-dependent manner and works as a negative regulator of mTORC1 during osteoclastogenesis. Our study highlights the importance of proper levels of mTORC1 activation in osteoclasts for the proper maintenance of healthy bone and suggests MYC/GADD34 axis as a potential target for mTORC1 regulation in bone diseases.

## Materials and methods

### Mice

Myeloid-specific MYC deficient mice (MYC^ΔM^) were obtained as described previously (Bae et al., 2017). Mice with osteoclast-specific deletion of TSC2 (TSC2^ΔOC^) were generated by crossing TSC2-floxed mice (Jackson Laboratory) with Cathepsin K-Cre mice (Nakamura et al., 2007), which were kindly provided by Dr. Matthew Greenblatt (Weill Cornell Medicine). Control WT mice consisted of littermate Cathepsin K-Cre mice for TSC2^ΔOC^ mice. All animals were maintained in a specific pathogen-free environment in the Weill Cornell Medicine College vivarium. All the experiments conformed to the ethical principles and guidelines approved by the Institutional and Animal Care and Use Committee of Weill Cornell Medical College.

### Analysis of bone phenotype

μCT analysis (Bouxsein et al., 2010) and histomorphometry analysis was performed as described previously (Bae et al., 2017). Femurs were scanned using a μCT, with an isotropic voxel resolution of 6 µm (μCT35, Scanco, Brüttisellen, Switzerland; 55 kVp, 145 μA, 600 ms integration time). For analysis of femoral bone mass, the volume of interest (VOI) encompassed a 200-slice section in the metaphysis, proximal to the growth plate. To ensure the exclusion of primary spongiosa in the growth plate, VOIs began 55 slices proximal to the distal end of the growth plate. Trabecular bone parameters included bone volume fraction (BV/TV), trabecular thickness (Tb. Th), trabecular separation (Tb. Sp), and trabecular number (Tb. N). Three-dimensional reconstructions were generated by stacking thresholded 2D images from the contoured region. All samples were included in the analysis conducted in a blinded manner. After μCT analysis was done, femurs were decalcified with 10% buffered EDTA (Sigma-Aldrich) and embedded in paraffin. To assess osteoclast or osteoblast visualization on trabecular bone within the femur metaphysis, sections were stained with tartrate-resistant acid phosphatase (TRAP) and methyl green or hematoxylin and eosin (Vector Laboratories, Burlingame, CA, United States). All measurements were performed using Osteometric software (Osteomeasure) according to standard procedures (Parfitt et al., 1987). Osteoclasts were identified as TRAP-positive cells that were multinucleated and adjacent to bone.

### Reagents

Human macrophage colony-stimulating factor (M-CSF) and RANKL was purchased from Peprotech (Rocky Hill, NJ, United States). Rapamycin was obtained from Calbiochem. Antibodies were used for immunoblotting as follows: NFATc1 (Santa Cruz Biotechnology; sc-7294); α-tubulin (Sigma-Aldrich; T9026); phosphorylated p70S6K, p70S6K (Cell Signaling Technology; 9234, 2708); and GADD34 (Proteintech; 10449-1-AP). A mouse CTX-I and P1NP ELISA kits were purchased from Cloud-Clone (Wuhan, China).

### Mouse osteoclast differentiation

Mouse bone marrow-derived macrophages (BMDMs) were prepared from bone marrow cells from 8-week-old mice as described previously (Bae et al., 2017). Non-adherent mouse bone marrow cells were cultured with α-MEM w/o nucleosides (Invitrogen) supplemented with 10% premium FBS (R&D systems), 1% L-glutamine (Invitrogen), and 1% penicillin/streptomycin (Invitrogen). For mouse osteoclast differentiation, mouse BMDMs were seeded on the 96-well plates at a density of 1 × 10^4^ cells per well in triplicate with human M-CSF (20 ng/ml). Next day, cells were stimulated with human RANKL (50 ng/ml) and cultured for 3 days. Fresh medium with cytokines were replenished at day 2. Cells were fixed and stained for TRAP using the acid phosphatase leukocyte diagnostic kit (Sigma-Aldrich) as directed in the kit manual. Multinucleated (more than 3 nuclei) TRAP-positive osteoclasts were counted in triplicate wells.

### Human osteoclast differentiation

Human CD14-postive cells were prepared from peripheral blood as described previously (Mun et al., 2021). Human CD14+ cells were cultured with α-MEM without nucleoside supplementation with 10% defined FBS (Cytiva) and 1% L-glutamine. For human osteoclast differentiation, human CD14-postive cells were plated on 96-well plates at a density of 1 × 10^5^ cells per well in triplicate with human M-CSF (20 ng/ml) followed by human RANKL stimulation (40 ng/ml). Cells were cultured for 3 days.

### Measurement of osteoclast activity

Mouse BMDMs or human CD14-positive cells were differentiated to mature osteoclasts as described above using Corning Osteo Assay Surface 96-well plates (Sigma-Aldrich). After rapamycin treatment for the indicated time points, cells were removed, and the remaining mineralized matrix and formed resorption pits were visualized with Toluidine Blue staining. The resorbed area was calculated as previously described (Fujii et al., 2021).

### RNA sequencing and analysis

Total RNA was extracted using the RNeasy Mini Kit with DNase treatment (QIAGEN). RNA was processed and analyzed as described previously (Bae et al., 2017). Data from 2 biological replicates were used for bioinformatic analysis. The Gene Set Enrichment Analysis (GSEA) was used to analyze differentially expressed genes (https://www.gsea-msigdb.org/gsea/). NES (normalized enrichment score) is the primary static for examining gene set enrichment results (http://software.broadinstitue.org/doc/GSEAUserGuideTEXT.html#_Enrichment_Score_(ES).

### Gene expression analysis

DNA-free RNA was obtained using the RNeasy Mini Kit with DNase treatment (QIAGEN), and 0.5 μg of total RNA was reverse transcribed using a First Strand cDNA Synthesis kit (Fermentas). Real-time quantitative PCR was performed with Fast SYBR Green Master Mix and a 7500 Fast Real-time PCR system (Applied Biosystems). Expression of the tested genes were normalized relative to the levels of *Hprt*. The primer sequences were as follows: murine *Ppp1r15a* (GADD34) Forward: 5′-GAC​CCC​TCC​AAC​TCT​CCT​TC-3′ and Reverse: 5′-CTT​CCT​CAG​CCT​CAG​CAT​TC-3′, *Hprt* Forward: 5′-TCC​TCA​GAC​CGC​TTT​TTG​CC-3′ and Reverse: 5′-CTA​ATC​ACG​ACG​CTG​GGA​CT-3′.

### Immunoblotting analysis

Whole-cell extracts were electrophoretically separated on 7.5% SDS-PAGE, transferred to polyvinylide fluoride membranes (Millipore) and probed with specific antibodies.

### RNA interference

Mouse BMDMs were transfected with 50 nmol of siRNA oligonucleotides (SMARTpool: ON-TARGETplus Ppp1r15a siRNA: L-047639-00-0005, ON-TARGETplus Non-targeting Pool: D-001810-10-20, Dharmacon) using the TransIT-TKO^®^ transfection reagent (Mirus Bio) according to the manufacturer’s instructions.

### Statistical analysis

All statistical analyses were performed with GraphPad Prism 8 (GraphPad Software) using a two-tailed, paired or unpaired *t* test for two conditions or one-way ANOVA for multiple comparisons with a Tukey’s *post-hoc* test. A *p* value of less than 0.05 was considered statistically significant.

## Results

### MYC is an upstream regulator of mammalian target of rapamycin complex 1 activation

**FIGURE 1:**
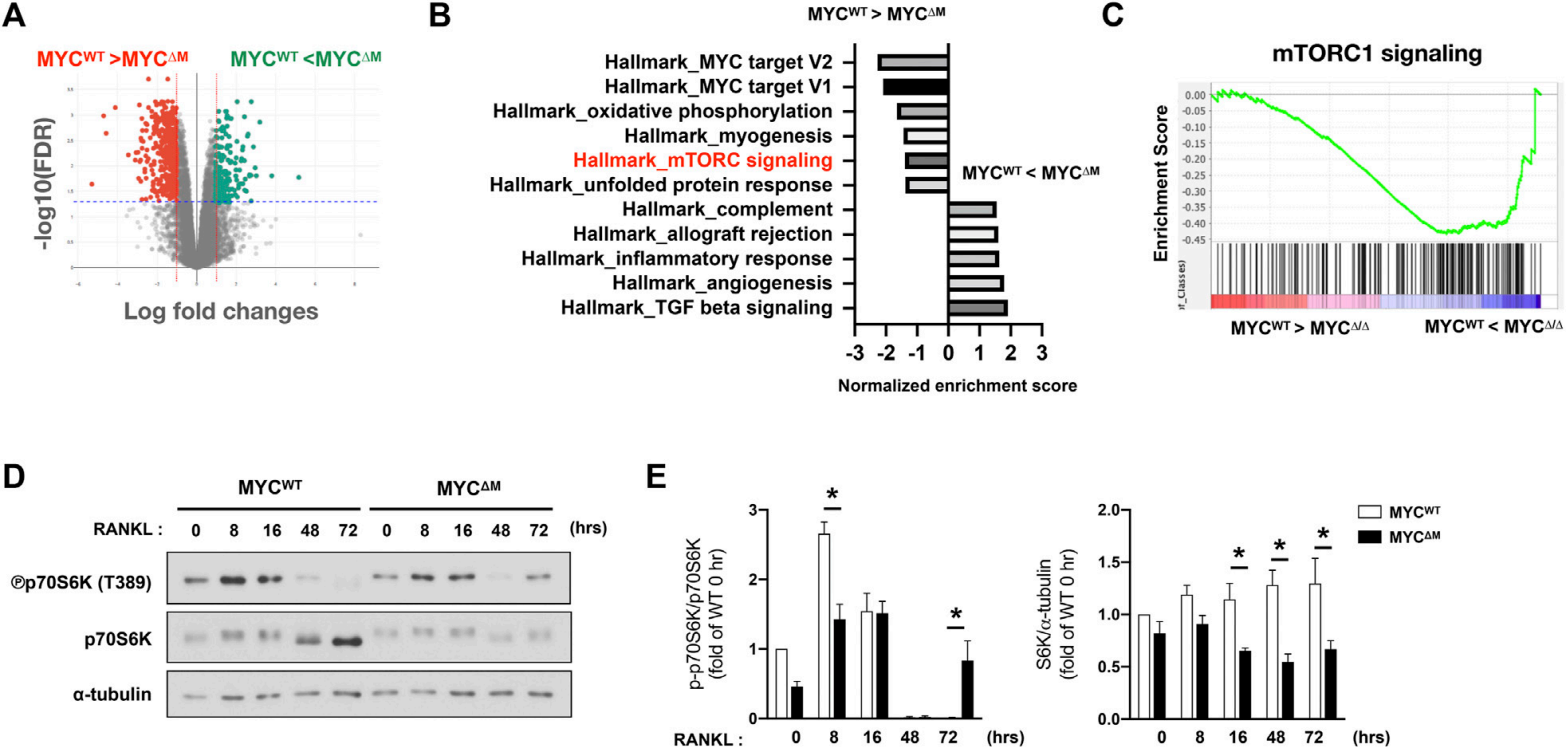
MYC regulates mTORC1 activity in osteoclastogenesis. **(A)** Mouse BMDMs from control (MYC^WT^) mice and MYC-deficient (MYC^ΔM^) mice were stimulated with RANKL for 1 day. Volcano plot of differentially expressed genes (DEGs) in MYC-deficient cells relative to control cells after RANKL stimulation for 24 h from the RNA sequencing (RNA-seq) analysis (>1.5 fold). **(B,C)** Gene-set–enrichment analysis of DEGs with genes ranked on the basis of expression in MYC^ΔM^ relative to that in MYC^WT^, showing the distribution of genes in the mTORC1 signaling gene set against the ranked list of the genes from the RNA-seq analysis. NES: normalized enrichment score, the primary statistic for examining gene set enrichment results. **(D)** Immunoblot of whole-cell lysates from control and MYC-deficient BMDMs after RANKL stimulation for the indicated time points with anti-phospho p70S6K, p70S6K, and α-tubulin antibodies. α-tubulin served as a loading control. Data are representative of four experiments. **(E)** Graph depicts the quantification of signal intensity of the phospho p70S6K and total p70S6K immunoblot using densitometry, normalized to WT control. Data are shown as mean ± SEM (*n* = 4). *p < 0.05 by one-way ANOVA with a *post hoc* Tukey test.

We and others have established MYC as a key regulator of osteoclastogenesis (Battaglino et al., 2002; Bae et al., 2017). MYC expression is induced by RANKL, and MYC deficiency has been shown to suppress osteoclast formation and its bone-resorbing activity (Bae et al., 2017). To gain insight into the downstream pathways that are regulated by MYC at the early phases of osteoclastogenesis, we performed an unbiased transcriptomic analysis using RNA-seq to identify genes whose expression was affected by MYC in the early phases of the RANKL response. Bone marrow cells from control LysM-Cre (MYC^wt^) and myeloid cell-specific MYC deficient (MYC^f/f^ LysM-Cre, named MYC^ΔM^) mice were differentiated into BMDMs and then cultured with M-CSF and RANKL for 24 h. 1,332 differentially expressed genes (DEGs, 1.5 fold changes, FDR < 0.05) between MYC^wt^ and MYC^ΔM^ cells were detected; 454 genes were upregulated, and 888 genes were downregulated in MYC-deficient cells (Figure 1A). We performed gene set enrichment analysis (GSEA) (Mootha et al., 2003; Subramanian et al., 2005), broadly testing for the enrichment of well-defined gene sets from the comprehensive Molecular Signature Data Base v5.1 (www.broadinstitute.org) of DEGs. GSEA pathway analysis of DEGs revealed that MYC regulates genes related to mTORC1 signaling (Figures 1B,C). To check if mTORC1 signaling is affected by MYC deficiency, control and MYC-deficient BMDMs were stimulated with RANKL, and mTORC1 activity was examined. The activation of mTORC1 signaling can be determined by detecting the phosphorylation of its downstream targets, ribosomal protein S6 Kinase (S6K) and 4EBP1 (Ma and Blenis, 2009); phosphoS6K was used to detect mTORC1 activation in this study. We found that the levels of mTORC1 activity differed at early and late stages of osteoclastogenesis. mTORC1 was activated at the early phases of the RANKL response but was suppressed during the later phases of osteoclastogenesis (Figures 1D,E). Intriguingly, the biphasic regulation of mTORC1 activity was dependent on MYC (Figures 1D,E); the early activation of mTORC1 and suppression of mTORC1 at the later phase of osteoclastogenesis were attenuated by MYC deficiency. In addition, the expression of total p70S6K expression was also diminished in MYC-deficient cells (Figures 1D,E). Thus, our results suggest that MYC is required for both the early activation and late suppression of mTORC1 activity.

### The level of mammalian target of rapamycin complex 1 activity affects osteoclast function

**FIGURE 2:**
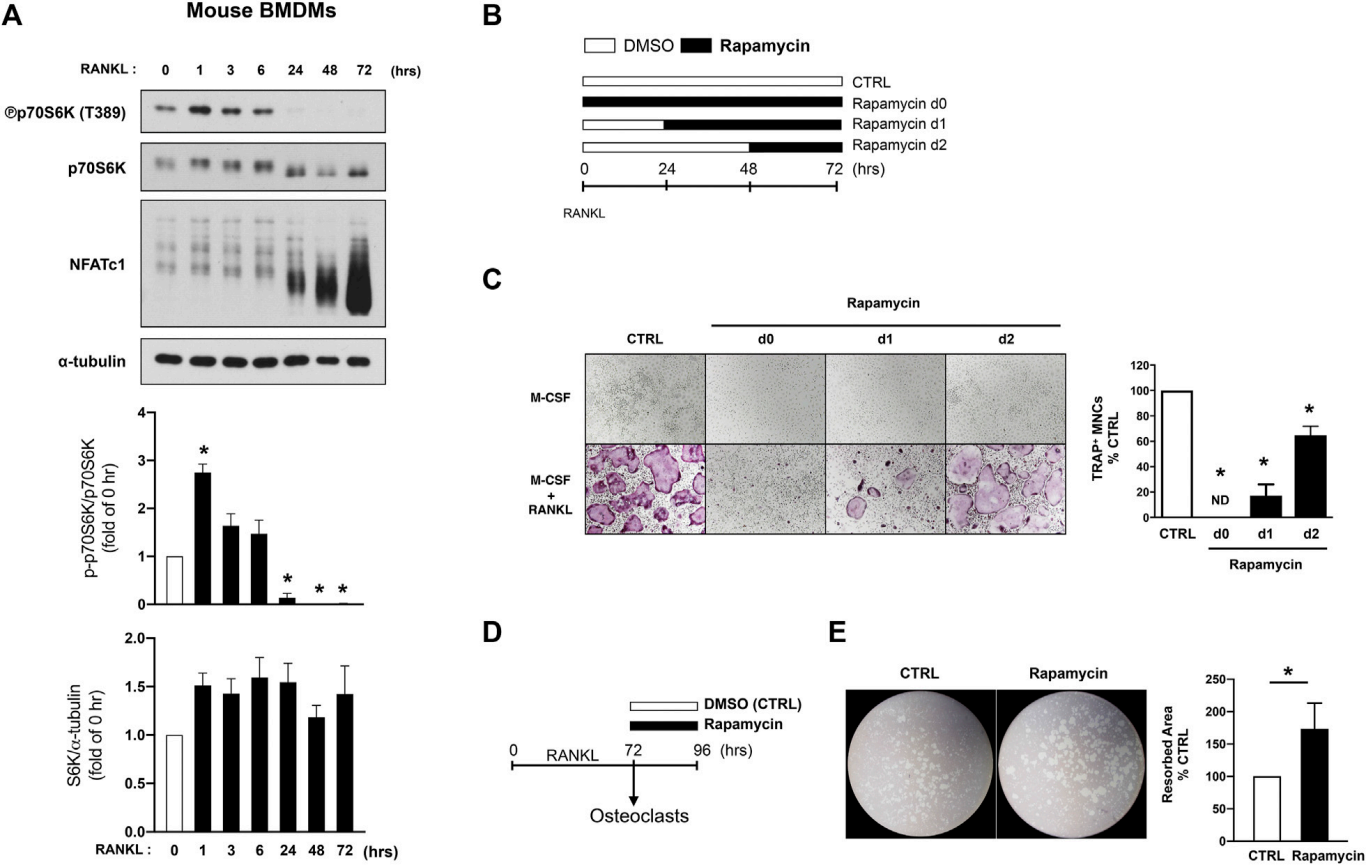
mTORC1 signaling has a biphasic effect on mouse osteoclastogenesis. **(A)** Immunoblot of whole-cell lysates from mouse BMDMs after RANKL stimulation for the indicated time points with anti-phospho p70S6K, p70S6K, NFATc1, and α-tubulin antibodies. α-tubulin served as a loading control. Graph depicts the quantification of signal intensity of the phospho p70S6K and total p70S6K immunoblot using densitometry, normalized to the control (*n* = 3). **(B)** A schematic diagram illustrating the experimental design in **(C)**. **(C)** Representative images show osteoclast differentiation after rapamycin treatment (100 nM) at different time points after RANKL stimulation. Graph depicts the number of TRAP-positive multinucleated cells (MNCs) counted in triplicate from three independent experiments. **(D)** A schematic diagram illustrating the experimental design in **(E)**. **(E)** Representative images show bone resorption activity of osteoclasts after rapamycin treatment for 24 h. Graph depicts the percentage of resorbed pit area per total area from six independent experiments. All data are shown as mean ± SEM. *p < 0.05 by one-way ANOVA with a *post hoc* Tukey test **(A,C)** or two-tailed, paired *t* test **(E)**. ND, not detected.

To further dissect the role of biphasic regulation of mTORC1 in osteoclastogenesis, we measured the regulation of mTORC1 activity in human and mouse cells. During mouse osteoclast differentiation, mTORC1 was activated 1 hour after RANKL stimulation and subsequently was suppressed below the baseline at 24 h after RANKL stimulation, as supported by a previous study (Hiraiwa et al., 2019) (Figure 2A). Given the transient activation of mTORC1 during osteoclastogenesis, we considered the possibility of a differential role of mTORC1 at different stages of osteoclastogenesis. To test this, we inhibited mTORC1 activation by rapamycin treatment, an inhibitor of mTORC1 (Li et al., 2014), at different time points after RANKL stimulation (Figure 2B). Consistent with the previous studies showing that mTORC1 activation is required for osteoclastogenesis (Glantschnig et al., 2003; Dai et al., 2017a), rapamycin treatment suppressed osteoclastogenesis (Figure 2C). While inhibition of mTORC1 by rapamycin treatment prior to RANKL stimulation nearly completely suppressed osteoclast formation, osteoclastogenesis was inhibited to a lesser extent when rapamycin was added at 2 days after RANKL stimulation (Figure 2C). We next tested if this suppressed mTORC1 activity is related to the bone-resorbing function of osteoclasts. Bone marrow-derived macrophages (BMDMs) were cultured with M-CSF and RANKL for 3 days to form mature osteoclasts, and mature osteoclasts then were treated with rapamycin or DMSO (Figure 2D). Notably, rapamycin-treated osteoclasts exhibited greater resorbing activity compared to vehicle-treated osteoclasts (Figure 2E). Rapamycin treatment on mature osteoclasts minimally affected TRAP-positive osteoclasts as displayed by TRAP-positive area, while significantly increasing resorbing area per TRAP-positive area (Supplementary Figure S1), suggesting that mTORC1 inactivation at the later stage of osteoclastogenesis may be required for the appropriate function of osteoclasts.

**FIGURE 3:**
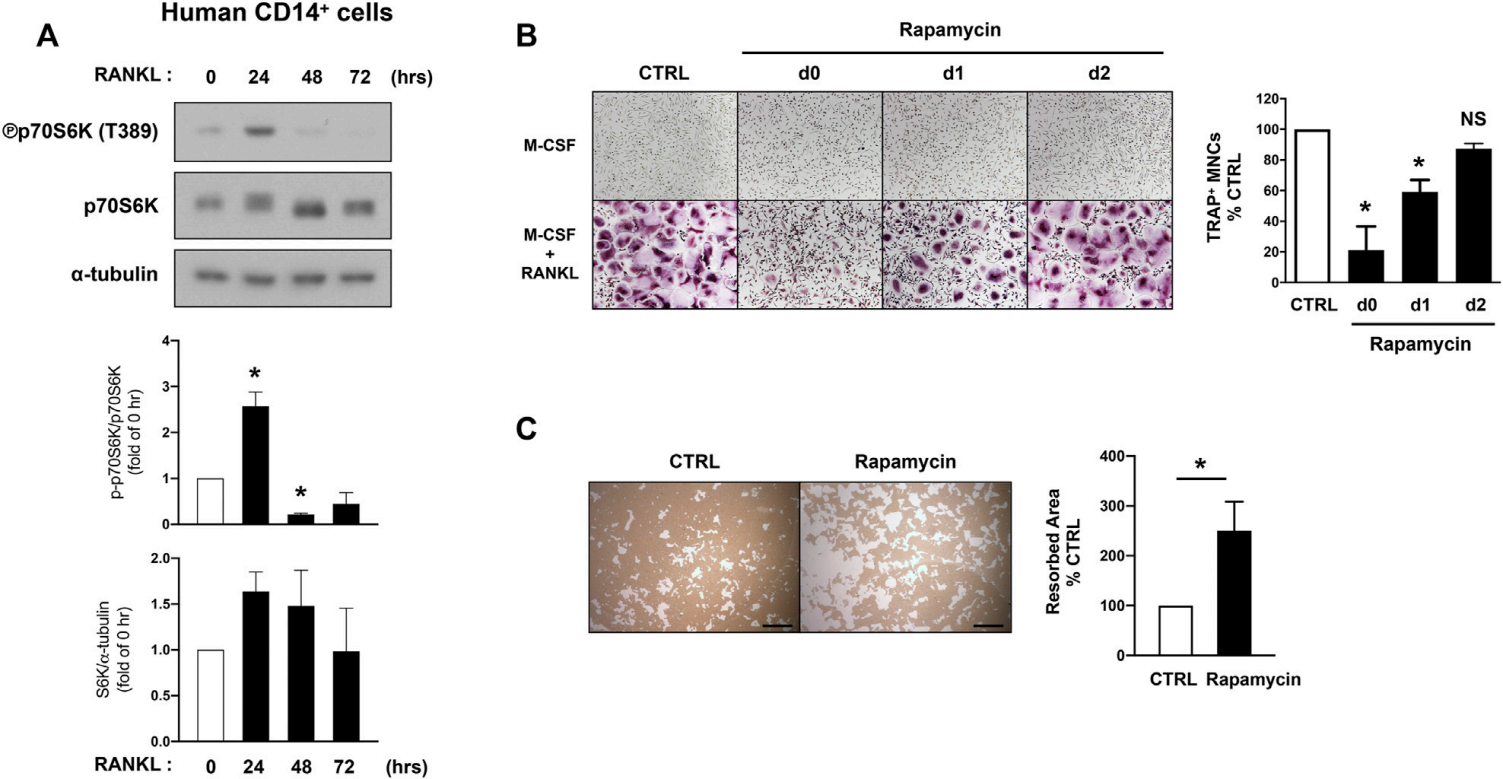
mTORC1 signaling has a biphasic effect on human osteoclastogenesis. **(A)** Immunoblot of whole-cell lysates from human CD14-positive cells after RANKL stimulation for the indicated time points with anti-phospho p70S6K, p70S6K, and α-tubulin antibodies. α-tubulin served as a loading control. Representative results from four independent donors are shown. Graph depicts the quantification of signal intensity of the phospho p70S6K and total p70S6K immunoblot using densitometry, normalized to the control (*n* = 4). **(B)** Representative images show human osteoclast differentiation after rapamycin treatment (100 nM) at different time points after RANKL stimulation. Graph depicts the number of TRAP-positive multinucleated cells (MNCs) counted in triplicate. Data are from four independent donors. **(C)** Representative images show bone resorption activity of human osteoclasts after rapamycin treatment for 48 h. Graph depicts the percentage of resorbed pit area per total area. Data are from four independent donors. Scale bar: 500 μm. All data are shown as mean ± SEM. *p < 0.05; NS, not significant by one-way ANOVA with a *post hoc* Tukey test **(A,B)** or two-tailed, paired *t* test **(C)**.

Hyperactive mTORC1 signaling is a clinical feature of patients with tuberous sclerosis complex (TSC) which is caused by inactivating pathogenic variants in either TSC1 or TSC2 genes, and sclerotic bone lesions have been frequently reported in TSC patients (Avila et al., 2010; Wu et al., 2017). However, the role of mTORC1 activity in human osteoclast differentiation has not been demonstrated yet. We examined how its activity is regulated in human osteoclastogenesis. Human CD14-postive cells were cultured with M-CSF and RANKL, and mTORC1 activity was examined by detecting the phosphorylation of S6K. Consistent with our observation in mouse cells, mTORC1 activity was dynamically regulated by RANKL stimulation; mTORC1 activity increased in response to RANKL at 24 h after stimulation, but its activity was decreased at the later phase of human osteoclastogenesis (Figure 3A). Consistent with the data observed in mouse cells (Figure 2), blocking mTORC1 activity by rapamycin treatment up to day one after RANKL stimulation suppressed osteoclast differentiation (Figure 3B). However, osteoclast differentiation was minimally affected when mTORC1 was inhibited at 2 days after RANKL stimulation (Figure 3B). After culturing human CD14-positive cells with M-CSF and RANKL to form mature osteoclasts, cells were treated with rapamycin or DMSO for 2 days. Furthermore, the suppression of mTORC1 in human mature osteoclasts by rapamycin treatment enhanced their bone-resorbing function (Figure 3C). Taken together, our results suggest that lower activity of mTORC1 at the later phases of osteoclastogenesis has a beneficial effect on the bone-resorbing function of osteoclasts.

### Activation of mammalian target of rapamycin complex 1 in osteoclasts *in vivo* dysregulates bone homeostasis

**FIGURE 4:**
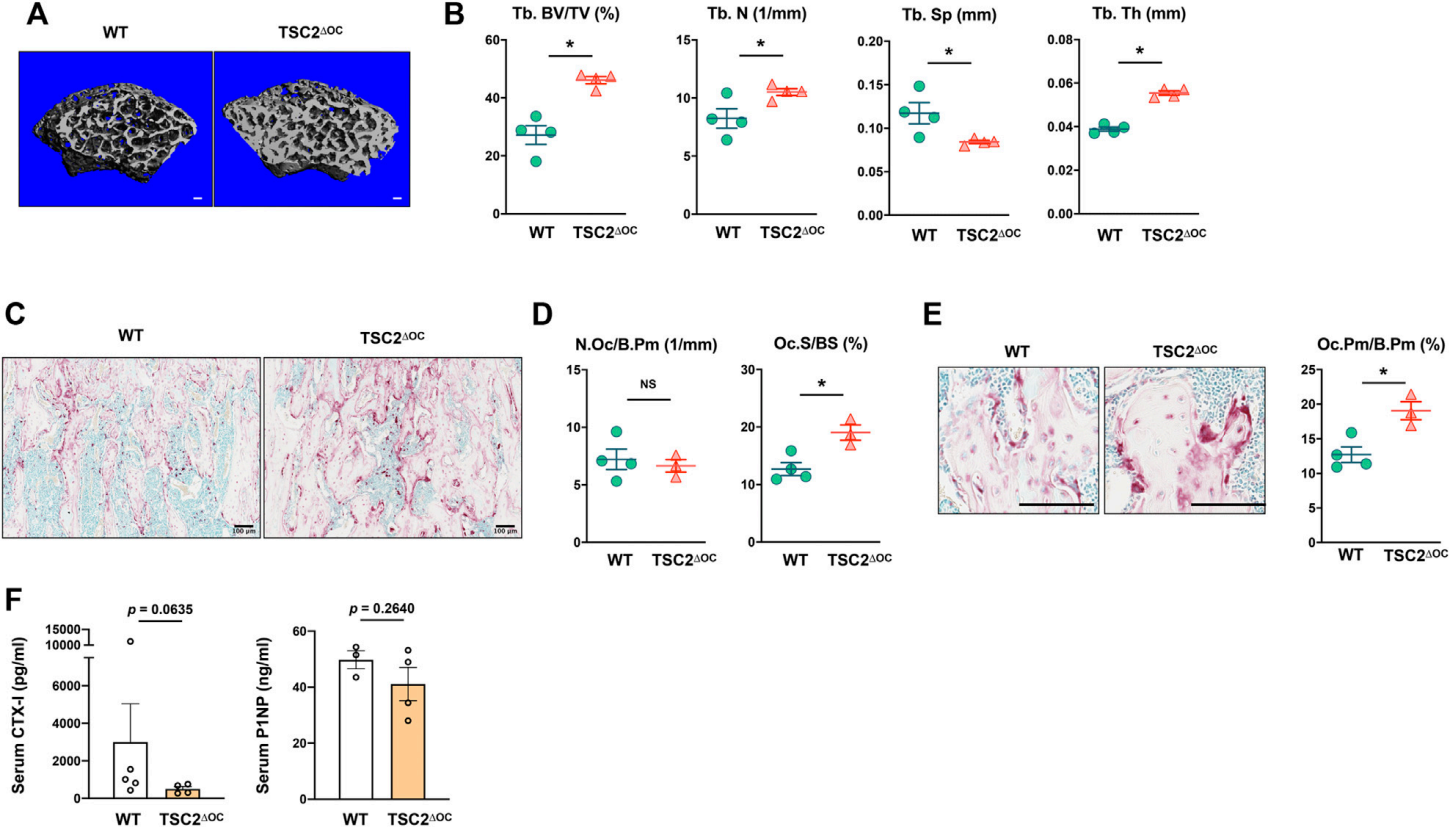
Hyperactivation of mTORC1 in osteoclasts impedes bone resorption. **(A)** Representative μCT reconstructed images of the trabecular architecture of the distal femurs from 8-week-old female TSC2-floxed Cathepsin K-Cre (TSC2^ΔOC^) and littermate control Cathepsin K-Cre (WT) mice. **(B)** μCT measurements for the indicated parameters of the trabecular bone in distal femurs. Bone volume/tissue volume ratio (BV/TV), trabecular numbers (Tb.N), trabecular thickness (Tb.Th), and trabecular space (Tb.Sp) were determined by μCT analysis. **(C)** Representative images showing the TRAP-positive, multinucleated osteoclasts (red) in the coronal sections of the distal femur. **(D)** Histomorphometric analysis of the trabecular bone. Number of osteoclasts per bone perimeter (N.Oc/B.Pm), osteoclast surface area per bone surface (Oc.S/BS). **(E)** Enlarged images from **(C)** showing the size of TRAP-positive, multinucleated osteoclasts (red). Graph depicts the size of osteoclasts. Osteoclast perimeter per bone perimeter (Oc.Pm/B.Pm). **(F)** The concentration of serum CTX-I and PINP measured by ELISA. All data are shown as mean ± SEM. *p < 0.05; NS, not significant by two-tailed, unpaired *t* test. Scale bar: 100 μm.

To corroborate our observation showing mTORC1 activity inversely regulates the bone-resorbing function of osteoclast, we wished to force mTORC1 activation at the late phase of osteoclastogenesis *in vivo*. Given that TSC1 or TSC2 deficiency results in a hyperactive mTORC1 state (Inoki et al., 2002; Huang and Manning, 2008), we generated TSC2-floxed CtsK-Cre (TSC2^∆OC^) mice by crossing TSC2-floxed mice with Cathepsin K-Cre mice and investigated their bone phenotype. Strikingly, TSC2^∆OC^ mice exhibited a significantly higher trabecular bone mass compared to wild-type (WT) mice (Cathepsin K-Cre), along with significantly increased number and thickness of trabecular bone and decreased trabecular spacing (Figures 4A,B). Histomorphometric analysis of trabecular bone revealed that the number of osteoclasts was not changed in TSC2^∆OC^ mice (Figures 4C,D). Similar to TSC1 deficient mice (Wu et al., 2017), TSC2^∆OC^ mice had larger osteoclasts compared to those from WT mice (Figures 4D,E). There was no difference in the number of osteoblasts or the level of N-terminal propeptide of type I procollagen (P1NP), a serum bone formation marker, between TSC2^∆OC^ mice and WT mice (Supplementary Figure S2; Figure 4F). Nonetheless, the level of CTX-I, a serum bone resorption marker, exhibited a decreasing trend in TSC2^∆OC^ mice compared to WT mice (Figure 4F), indicating that osteoclasts from TSC2^∆OC^ mice exhibited an attenuated resorbing function and caused increased bone mass. Taken together, using loss-of-function and gain-of function studies suggested that lower mTORC1 activity in mature osteoclasts is required for osteoclastic bone resorption.

### The MYC/GADD34 axis negatively regulates mammalian target of rapamycin complex 1 in osteoclast differentiation

We wished to determine the mechanism of mTORC1 inactivation in the late phase of osteoclastogenesis by MYC. To search for the upstream regulator responsible for repressing mTORC1, we screened known regulators of mTORC1, including Deptor (Varusai and Nguyen, 2018), Ddit4 (Foltyn et al., 2019), FOXOs (Lin et al., 2014), and BNIP3 (Li et al., 2007) on mTORC1 activation. However, none of these were able to rescue suppressed mTORC1 activity (data not shown).

**FIGURE 5:**
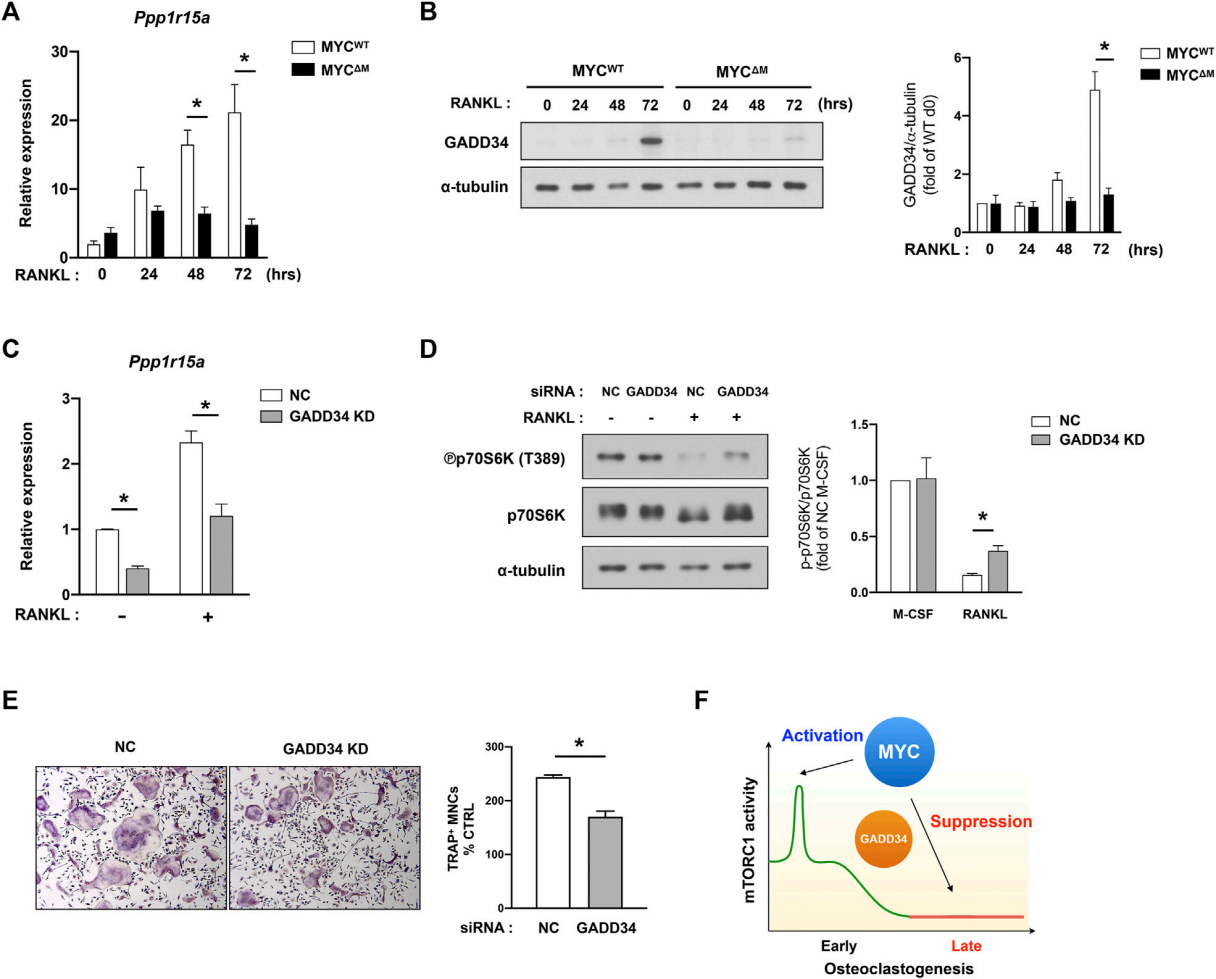
MYC/GADD34 axis inactivates mTORC1 in osteoclasts. **(A)** The mRNA expression of *Ppp1r15a* (encodes GADD34, relative to the *Hprt* housekeeping gene) in control (MYC^WT^) and MYC-deficient (MYC^ΔM^) BMDMs after RANKL stimulation for the indicated time points (*n* = 4). **(B)** The protein expression of GADD34 determined by immunoblot using whole-cell lysates of control and MYC-deficient BMDMs after RANKL stimulation for the indicated time points. α-tubulin served as a loading control. Data are representative of three independent experiments. Graph depicts the quantification of signal intensity of the GADD34 immunoblot using densitometry and then normalized to WT control (*n* = 3). **(C–E)** Mouse BMDMs were transfected with siRNAs specific for GADD34. **(C)** The mRNA expression of *Ppp1r15a* (relative to the *Hprt* housekeeping gene) in negative-control (NC) or siRNAs specific for GADD34 transfected cells after RANKL stimulation for 3 days (*n* = 3). **(D)** Representative images for Immunoblot of whole-cell lysates with indicated antibodies. α-tubulin served as a loading control. Representative results from four independent experiments are shown. Graph depicts the quantification of signal intensity of the phospho p70S6K immunoblot using densitometry and taken as a ratio of phospho p70S6K over total p70S6K and then normalized to NC control (*n* = 4). **(E)** Osteoclastogenesis assay. BMDMs were cultured with M-CSF and RANKL for 3 days. Left, Representative images of TRAP-stained cells. Right, Quantification of TRAP positive multinucleated (more than three nuclei) cells (*n* = 3). **(F)** A diagram illustrating the dynamic regulation of mTORC1 activity by MYC. All data are shown as mean ± SEM. *p < 0.05 by one-way ANOVA with a *post hoc* Tukey test **(A–D)** or by two-tailed, paired *t* test **(E)**.

To search for additional candidates, we referenced the RNA-seq analysis of MYC-deficient cells and found that the expression of genes associated with the unfolded protein response (UPR) were diminished in MYC-deficient cells (Figure 1); this was of particular interest given that the balance between the mTORC1 pathway and UPR tightly regulates protein synthesis (Walter and Ron, 2011). Growth arrest and DNA damage-inducible protein (GADD34, encoded by *Ppp1r15a*) is an important factor for the coordinate regulation of UPR. Moreover, in 293HEK cells and mouse embryonic fibroblasts, GADD34 leads to dephosphorylation of TSC2 and negatively regulates mTOR signaling (Watanabe et al., 2007; Uddin et al., 2011). Since GADD34 regulation in osteoclasts had not yet been investigated, we first tested GADD34 expression in osteoclastogenesis. Control and MYC deficient BMDMs were treated with RANKL, and the expression of GADD34 was measured. The transcription level of Gadd34 (*Ppp1r15a*) was increased in WT cells upon RANKL stimulation in a time-dependent manner, while MYC-deficient cells failed to induce its expression (Figure 5A). Concomitantly, RANKL-induced GADD34 protein expression was abrogated by MYC deficiency (Figure 5B), indicating MYC regulates the expression of GADD34 in osteoclastogenesis. To test if GADD34 plays a role in regulation of mTORC1 activity in osteoclasts, we knocked down the expression of GADD34 using nucleofecting siRNA oligos against *Ppp1r15a* and evaluated mTORC1 activity. *Ppp1r15a* mRNA expression was efficiently diminished in Gadd34-deficient cells compared to control cells (Figure 5C). GADD34 deficiency partially restored RANKL-induced mTORC1 inactivation (Figure 5D), suggesting that the MYC/GADD34 axis, in part, contributes to the suppression of mTORC1 in osteoclasts. GADD34 deficiency also suppressed osteoclastogenesis (Figure 5E). GADD34 deficiency showed a decreasing trend in osteoclastic bone resorption (Supplementary Figure S3). Taken together, our results suggest that GADD34 is a positive regulator of osteoclastogenesis and suppresses mTORC1 activity at the later stages of osteoclastogenesis.

## Discussion

Both the mTOR pathway and MYC are critical modulators of osteoclastogenesis (Park-Min, 2019). However, the crosstalk between MYC and the mTOR pathway during osteoclastogenesis has never been studied previously. In this study, we show that MYC is an upstream regulator of mTOR pathway and modulates a biphasic activation of mTORC1 during osteoclastogenesis. It is well-documented that early activation of mTORC1 is required for osteoclast formation (Dai et al., 2017a; Zhang et al., 2017; Hiraiwa et al., 2019). However, we found that inactivation of mTORC1 in mature osteoclasts is important for the bone-resorbing function of osteoclasts. Accordingly, hyperactivation of mTORC1 in osteoclasts, by deleting TSC2 in mice, increased bone mass and suppressed osteoclast activity. MYC promoted mTORC1 activation at the early phases of osteoclastogenesis and suppressed mTORC1 activation at the later phases of osteoclastogenesis *via* induction of GADD34 (Figure 5F). Overall, our findings suggest that MYC regulates osteoclast differentiation and activity by tightly controlling mTORC1 activity.

MYC is a multifunctional helix-loop-helix leucine zipper transcription factor (Dang, 2012). Our previous report established the central role of MYC in osteoclasts and pathological bone loss. MYC is induced by RANKL stimulation (Bae et al., 2017). MYC is required for the induction of NFATc1 and plays a key role in oxidative phosphorylation in osteoclasts (Park-Min et al., 2014; Bae et al., 2017). This study further suggests that MYC is an upstream regulator of mTORC1 signaling in osteoclasts. Despite the importance of mTORC1 in skeletal development, the mechanism underlying the dynamic regulation of mTORC1 activity in osteoclasts remains unclear. Here, we show that MYC deficiency not only inhibited RANKL-induced mTORC1 activation and the expression of p70S6K at the early phases of osteoclastogenesis but also activated mTORC1 activation at the late phases of osteoclastogenesis. RANKL-mediated mTORC1 activation can be regulated by other signaling pathways. It has been shown that mTORC1 is activated by PI3K (The phosphoinositide 3 kinase)/Akt *via* inhibition of TSC1/2 (Shaw and Cantley, 2006). The PI3KAkt/mTOR pathway plays an important role in osteoclastogenesis (Moon et al., 2012) by several different mechanisms (Tiedemann et al., 2017; Yu et al., 2019). The mTOR-rictor-Akt circuit regulates osteoclast fusion and growth (Tiedemann et al., 2017). However, we did not detect any changes in the P13K/Akt pathway in MYC deficient cells (data not shown). In addition to the transcriptional control of osteoclast-specific genes by MYC, our study suggested that MYC may be involved in RANKL-induced protein synthesis in osteoclasts by regulating mTORC1 activity. Therefore, our results provide new insights into the role of MYC in osteoclastogenesis.

mTORC1 promotes anabolic functions including protein and lipid synthesis, and its activation is dependent on energy status and nutrient availability (Saxton and Sabatini, 2017). Aberrant mTORC1 signaling is associated with many human diseases, including cancer, diabetes, and neurological disorders (Inoki et al., 2005; Saxton and Sabatini, 2017). mTORC1 activation is also important for bone remodeling by regulating osteoblasts (Riddle et al., 2014; Chen and Long, 2015; Dai et al., 2017b; Fitter et al., 2017; Martin et al., 2018) and osteoclasts (Dai et al., 2017a; Zhang et al., 2017; Huynh and Wan, 2018). During osteoclastogenesis, mTORC1 activation is biphasically regulated. However, the role of mTORC1 in osteoclasts is still controversial. Pharmacological studies established that mTOR signaling positively regulates osteoclastogenesis. However, recent genetic studies demonstrated that constitutive active mTOR signaling in TSC1 or TSC2 deficient cells suppresses osteoclastogenesis regardless of the stage of osteoclastogenesis (Wu et al., 2017; Zhang et al., 2017). We also found that prolonged mTORC1 activation in TSC2^ΔOC^ mice increases bone mass. Osteoclasts from TSC2^ΔOC^ mice were larger, although the number of osteoclasts in TSC2^ΔOC^ mice was comparable to that of the control mice. In addition, cathepsin K-expressing osteoclasts in TSC2^ΔOC^ mice were bigger and showed increased phospho-mTOR compared to osteoclasts in control mice (data not shown). These results implicate the broad but complicated effect of mTORC1 on osteoclast differentiation and activity.

The phenotype of osteoclasts in TSC2^ΔOC^ mice was similar to the phenotype of osteoclasts in TSC1^f/f^-Ctsk-Cre mice (Wu et al., 2017). TSC1^f/f^-Ctsk-Cre mice exhibited high bone mass and reduced bone resorption (Wu et al., 2017; Xu et al., 2018). However, inhibition of mTORC1 activation by raptor deficiency at the late phase of osteoclastogenesis using Cathepsin K-Cre also resulted in higher bone mass than in control mice (Dai et al., 2017a). Since either hyperactive or hypoactive mTORC1 at the late phase of osteoclastogenesis resulted in inhibition of osteoclast formation and increased bone mass, it is hard to draw a conclusion based on the genetic modification. Here, we show that rapamycin treatment in mature osteoclasts enhanced bone-resorbing activity, suggesting the importance of inactive mTOR signaling at the late phase of osteoclastogenesis for osteoclastic bone resorption. While several studies demonstrated that rapamycin administration increased bone mass (Chen et al., 2015; Luo et al., 2016; Bateman et al., 2019), other studies showed that bone marrow cells isolated from mice after two month-treatment with low doses of rapamycin exhibited increased osteoclastogenic activity and diminished bone mass (Rubert et al., 2015; Huynh and Wan, 2018). However, since rapamycin targets not only osteoclasts but also osteoblasts and other cells, the outcome of rapamycin administration *in vivo* could result from the coordinated interaction among various cell types. Nonetheless, these studies support that selectively modulating mTORC1 activity could be used to control osteoclast differentiation and function in pathological conditions. Given that dysregulation of mTORC1 is important for the proper regulation of osteoclastogenesis, further studies dissecting the role of mTORC1 in physiological bone remodeling will be required.

Our study is the first to show the regulation of GADD34 in osteoclastogenesis. GADD34 is an important component of the unfolded protein response (UPR) and is quickly induced in nutrient- or amino acid- deprived conditions (Novoa et al., 2001; Gambardella et al., 2020). Although cellular energy deficiencies usually lead to UPR (Bravo et al., 2013), we show that GADD34 is gradually induced by RANKL stimulation even in nutrient-rich media. Moreover, ATP formation is greatly increased during osteoclastogenesis (Nishikawa et al., 2015; Bae et al., 2017), and adenosine monophosphate-activated protein kinase (AMPK), a cellular energy censor, is suppressed in osteoclasts (Bae et al., 2017, unpublished observations), suggesting that osteoclast formation is accompanied by increased energy metabolism. However, a discrepancy between GADD34 mRNA and protein expression during osteoclastogenesis has been observed. GADD34 mRNA was induced at 24 h after RANKL stimulation, while GADD34 protein gradually increased from 2 days after RANKL stimulation. GADD34 protein is remarkably unstable due to proteasomal degradation at steady-state conditions (Brush and Shenolikar, 2008). Our data suggests that GADD34 protein is stabilized by an unknown mechanism at the later phase of osteoclastogenesis. Furthermore, we also show that, upon RANKL stimulation, GADD34 was not induced in MYC-deficient cells, suggesting that MYC positively regulates the expression of GADD34 or the stabilization of GADD34 proteins. In contrast to our observations, Amundson et al. (1998) showed that stress-induced GADD34 was attenuated by overexpression of v-MYC. Given that RANKL-induced GADD34 expression is dependent on MYC in osteoclasts in nutrient-sufficient conditions, further study is needed to identify how MYC regulates GADD34 in osteoclasts. Collectively, our data show that both MYC deficiency and GADD34 deficiency reversed suppressed mTORC1 activity at the late phase of osteoclastogenesis, suggesting the inhibitory role of the MYC/GADD34 axis in mTORC1 activation in osteoclasts. Considering that MYC is currently undruggable (Prochownik and Vogt, 2010) and that mTORC1 is difficult to productively target due to its complicated function in osteoclastogenesis, the MYC/GADD34 axis could be a potential druggable therapeutic target controlling the abnormal activity of osteoclasts.

## Data Availability

The RNA-seq data have been deposited in the Gene Expression Omnibus database with the accession code GEO: GSE202932.
