# Supplementary material for: The crosstalk between MYC and mTORC1 during osteoclastogenesis
Source: Front Cell Dev Biol. 2022 Aug 19;10:920683. doi: 10.3389/fcell.2022.920683 (PMC9437285; doi:10.3389/fcell.2022.920683)
Supplement: Supplementary file 1 [file DataSheet1.pdf]

Supplementary Materials

The crosstalk between MYC and mTORC1 during osteoclastogenesis

Seyeon Bae, Brian Oh, Jefferson Tsai, Peter Sang Uk Park, Matthew Blake Greenblatt, Eugenia G Giannopoulou, and Kyung-Hyun Park-Min

Supplementary figure legend 2

Supplementary figures 1-3 3-5

Supplementary figure legend

Supplementary figure 1. The effect of rapamycin on the bone-resorbing activity of osteoclasts. Mouse BMDMs were differentiated to osteoclasts in the presence of M-CSF and RANKL using calcium-coated plates, as described in the Method section. Rapamycin (100 nM) was added to osteoclasts. After 24 hours, cells were stained with tartrate-resistant acid phosphatase (TRAP) to visualize osteoclasts, and then TRAP-positive area was quantified by Image J software. Additionally, the cells were removed, and the remaining mineralized matrix and formed resorption pits were visualized with Toluidine Blue staining. The resorbed area was calculated as described in the Method section. (A) Graph depicts the percentage of TRAP-positive area per total area. (B) Graph depicts the percentage of resorbed pit area per TRAP-positive area. Data are shown as mean ± SEM (n=3). *p< 0.05; NS, not significant by two-tailed, paired t test.

Supplementary figure 2. Osteoblast parameters in TSC2-floxed Cathepsin K-Cre ($TSC2^{\Delta OC}$) and WT mice. (A) Representative images showing the osteoblasts in the coronal sections of the distal femur. Scale bar: 50 μm. (B) Histomorphometric analysis of the trabecular bone. Graph depicts the number of osteoblasts per bone perimeter (Ob.N/B.Pm) and osteoblast surface area per bone surface (Ob.S/BS). Data are shown as mean ± SEM. NS, not significant by two-tailed, unpaired t test.

Supplementary figure 3. The effect of GADD34 on bone-resorbing activity of osteoclasts. Mouse BMDMs transfected with siRNAs specific for GADD34 or negative-control (NC) were differentiated to osteoclasts in the presence of M-CSF and RANKL using calcium-coated plates. (A) Representative images show bone resorption activity of GADD34-deficient osteoclasts. (B) Graph depicts the percentage of resorbed pit area per total area. Data are shown as mean ± SEM (n=4). P value was obtained by two-tailed, paired t test.

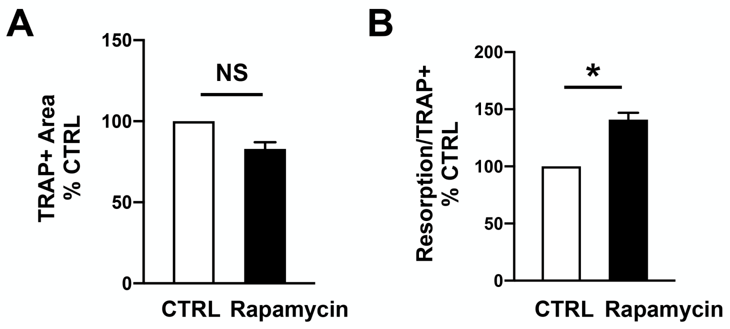

Supplementary Figure 1

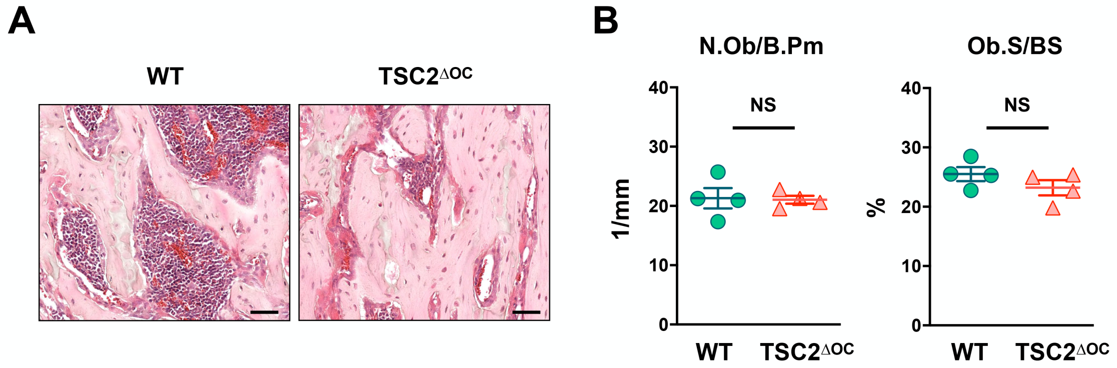

Supplementary Figure 2

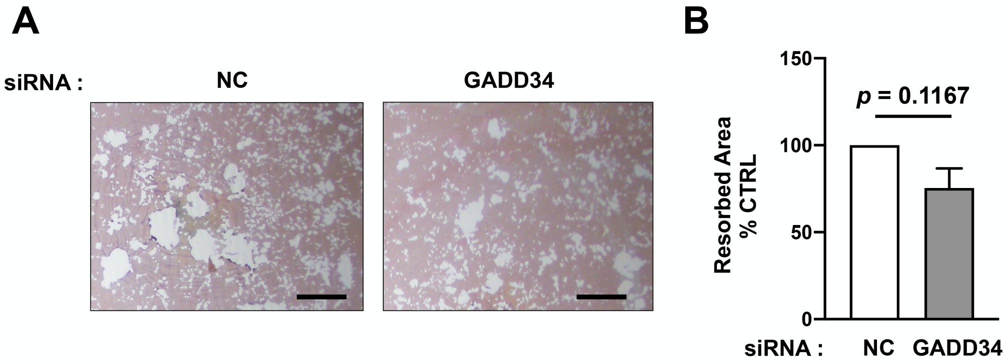

Supplementary Figure 3
